# Current challenges facing the assessment of the allergenic capacity of food allergens in animal models

**DOI:** 10.1186/s13601-016-0110-2

**Published:** 2016-06-16

**Authors:** Katrine Lindholm Bøgh, Jolanda van Bilsen, Robert Głogowski, Iván López-Expósito, Grégory Bouchaud, Carine Blanchard, Marie Bodinier, Joost Smit, Raymond Pieters, Shanna Bastiaan-Net, Nicole de Wit, Eva Untersmayr, Karine Adel-Patient, Leon Knippels, Michelle M. Epstein, Mario Noti, Unni Cecilie Nygaard, Ian Kimber, Kitty Verhoeckx, Liam O’Mahony

**Affiliations:** National Food Institute, Technical University of Denmark, Søborg, Denmark; TNO, Zeist, The Netherlands; Warsaw University of Life Sciences, Warsaw, Poland; Department of Bioactivity and Food Analysis, Institute for Food Science Research (CIAL) (CSIC-UAM), Madrid, Spain; INRA, Nantes, France; Nestlé Research Center, Lausanne, Switzerland; Institute for Risk Assessment Sciences, Utrecht University, Utrecht, The Netherlands; Food and Biobased Research, Wageningen University and Research Centre, Wageningen, The Netherlands; Department of Pathophysiology and Allergy Research, Medical University of Vienna, Vienna, Austria; UMR-INRA-CEA, Service de Pharmacologie et d’Immunoanalyse, Université Paris-Saclay, Gif-sur-Yvette, France; Danone Nutricia Research, Utrecht, The Netherlands; Utrecht Institute for Pharmaceutical Sciences, Utrecht University, Utrecht, The Netherlands; Experimental Allergy Laboratory, DIAID, Department of Dermatology, Medical University of Vienna, Vienna, Austria; Institute of Pathology, University of Bern, Bern, Switzerland; Norwegian Institute of Public Health, Oslo, Norway; University of Manchester, Manchester, UK; Swiss Institute of Allergy and Asthma Research, University of Zürich, Obere Strasse 22, 7270 Davos Platz, Switzerland

**Keywords:** Food allergy, Animal models, Novel allergens, Hazard identification

## Abstract

Food allergy is a major health problem of increasing concern. The insufficiency of protein sources for human nutrition in a world with a growing population is also a significant problem. The introduction of new protein sources into the diet, such as newly developed innovative foods or foods produced using new technologies and production processes, insects, algae, duckweed, or agricultural products from third countries, creates the opportunity for development of new food allergies, and this in turn has driven the need to develop test methods capable of characterizing the allergenic potential of novel food proteins. There is no doubt that robust and reliable animal models for the identification and characterization of food allergens would be valuable tools for safety assessment. However, although various animal models have been proposed for this purpose, to date, none have been formally validated as predictive and none are currently suitable to test the allergenic potential of new foods. Here, the design of various animal models are reviewed, including among others considerations of species and strain, diet, route of administration, dose and formulation of the test protein, relevant controls and endpoints measured.

## Background

**Fig. 1:**
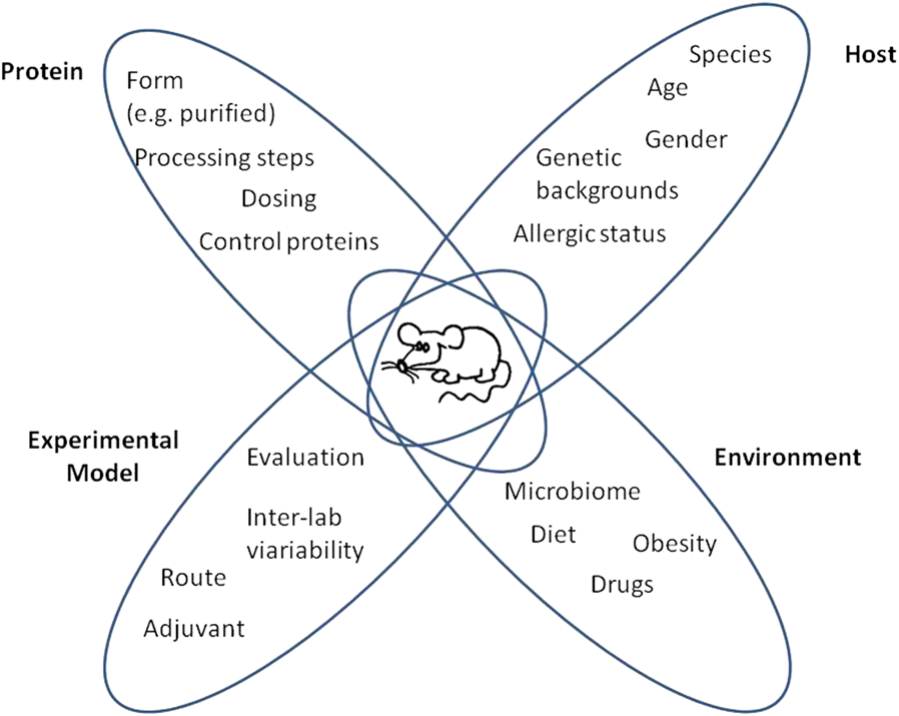
Factors which influence animal model design. Important considerations in the design, conduct and interpretation of animal models for assessment of the allergenic potential of food proteins are shown

Food allergy affects a significant proportion of the population and is associated with important health effects. In addition, food allergy has an impact on quality of life and represents a substantial economic burden [1, 2]. The exponential growth of the human population means that existing protein sources, such as soy, are being consumed by a wider population, while novel protein sources, such as insect and algae, are currently being examined for inclusion in human foodstuffs. The introduction of new proteins into the diet inevitably creates a potential opportunity for the development of new food allergies. There is a need, therefore, for the development and application of appropriate strategies for evaluating the allergenic potential of existing and new food proteins as an important component of safety assessment. A crucial question in food allergy research is what characteristics confer on proteins the ability to cause sensitization and allergy. Current understanding of this is incomplete and this has limited the development of predictive methods based on in silico analysis of protein sequence and structure, and in vitro methods most often based on the measurement of a single parameter. For this reason, there is a continued interest in the development of suitable animal models that provide a more holistic approach to the assessment of the allergic potential of proteins. Although there is a variety of animal models for evaluating allergenicity, none of the existing models has been validated, is predictive, or widely accepted [3]. Because the choice of animal experimental design as well as the selection of appropriate endpoints and evaluation parameters may lead to contradictory results, there is an enormous impact on performance and predictive accuracy of animal models. Here, we review the experimental design and interpretation of animal models for the assessment of the allergenic potential of novel food proteins (Fig. 1).

### Reference proteins

To assess the relative allergenicity of novel proteins, it is essential to use known allergens in humans as reference proteins. It is unknown why certain proteins are allergenic, compared to the large majority of non-allergenic food proteins. As reviewed extensively elsewhere [4], most plant allergens belong to the prolamin superfamily, including the lipid transfer proteins (LTP) and 2S albumins or to the cupin superfamily, which include the 11S and 7S globulins. Animal food allergens predominately belong to the parvalbumin, tropomysin and casein protein families. The shared conserved structure and biological activity among proteins in these families contribute to their allergenicity. However, protein homology alone does not guarantee allergenicity [5]. Sensitizing rats with 7S globulins from peanut, hazelnut, soy or pea induced IgE with different biological activities [6]. In addition, patients allergic to goat’s milk, but who tolerate cow’s milk, show an absence IgE-binding to bovine b-casein by IgE specific to caprine b-casein, despite a sequence identity of 91 % between the respective proteins [7]. This suggests that subtle differences in physical or biological properties are modulators of allergic responses even to well-described food allergens.

Initial studies by Astwood et al. [8] proposed that stability of proteins to in vitro gastric digestion significantly discriminated known allergens from non/low-allergens. However, subsequent studies with a wider range of proteins did not support these findings [9, 10]. Thus, additional tests, including those in animal models, may be useful in the assessment of the allergenic potential of novel proteins. Dearman and Kimber [11] showed that known allergenic proteins (peanut agglutinin and ovalbumin (OVA)) induced specific IgE upon intraperitoneal (i.p.) injection of mice, while presumed non/low-allergenic proteins (potato agglutinin, potato acid phosphatase) were immunogenic, but induced only low IgE titer responses. In contrast, a multi-laboratory study was unable to accurately differentiate between known allergens and putative non/low-allergens, including spinach rubisco and soy lipoxygenase [12]. Oral exposure to allergens under specific experimental conditions was able to distinguish allergenic from non/low-allergenic food extracts, while systemic exposure did not [13].

There is a significant need to validate a toolbox of reference proteins, which contain potent allergenic, weak and non/low-allergenic proteins. Published data, to date, demonstrate a lack of reproducible and predictive measurements, which emphasizes the need for in vivo models, that are thoroughly tested with a wide range of well-characterized, purified, and endotoxin-free potent, weak or non/low-allergenic proteins.

### Animal species and strain

The species most commonly employed in food allergy research is the mouse. In addition to their small size and short breeding cycle, the sequence of immunological events involved in the development of sensitization and the elicitation of allergic reactions is similar, although not identical to humans [14]. Moreover, the availability of various immunological and molecular reagents and transgenic animals makes them a powerful tool for investigating immunological mechanisms related to food allergies and evaluating the sensitizing potential of new food proteins.

As for humans, genetic predisposition in mice is important for measuring in vivo sensitization to novel food proteins. Smit et al. [15] examined three different murine strains following oral administration of peanut extract. Higher concentrations of peanut-specific IgE were found in BALB/c mice compared with other strains. In contrast, Berin et al. [16] reported no differences between BALB/c and C3H/HeOuJ mice in their IgE response to β-lactoglubulin (BLG) and C3H/HeOuJ mice produced higher peanut protein-specific IgE levels. These disparate results were attributed to the use of different protocols for inducing sensitization. Both Berin et al. [16] and Smit et al. [15] reported that spleen cells from peanut sensitized BALB/c mice secreted more IL-4, IL-5, IL-13 and IFN-γ than those from C3H/HeOuJ mice, suggesting that BALB/c mice might be the preferable strain.

As an alternative to the mouse, Brown Norway (BN) rats mount strong IgE antibody responses and due to their size, it is possible to monitor kinetics of serum specific antibody responses within individual animals. Another advantage is that the test protein can be delivered by daily gavage over a period of weeks in the absence of adjuvant [17]. On the downside, oral dosing of rats requires a larger amount of protein, compared to mice, which influences the cost of the experiment and fewer immunological reagents are available than for mouse.

In contrast to murine models, dogs spontaneously develop allergies [18]. Thus, it is a good species for evaluating food allergy. Moreover, it is also possible to immunize the same animal with multiple allergens. Furthermore, it is possible to do repeated endoscopy of the gut, to identify high IgE responder animals and their larger organ size and blood volume allows for more analyses and longitudinal studies. Although dogs are well suited for mechanistic studies, it is not feasible to use them for routine testing for safety assessment. They are expensive to maintain, there are a limited number of strains, they have greater inter-animal variation than rodents, and commercially available immunological reagents are lacking [17]. Similar drawbacks are present in swine with the addition of long dosing times needed for sensitization [12].

Other potential animal species are guinea pigs and rabbits. However, guinea pigs do not produce IgE. Allergic responses in guinea pigs are IgG1a mediated and possibly other mechanisms are also involved, thereby making the translation to humans more difficult. Rabbits generate high levels of IgE after subcutaneous sensitization, but are poorly characterized and thereby rarely used as a model species for food allergy [19].

In conclusion, mice are currently the most commonly used in vivo model for evaluating potential sensitizing capacities of food proteins. Notably, when using mouse models, it is important to wisely select an optimal strain and sensitization protocol, depending on the allergen source and specific research question.

## Route of sensitization

There are multiple routes used to induce allergic sensitization to food allergens including i.p., oral, intranasal (i.n.) and cutaneous administration [20, 21]. However, the route of administration may alter the resulting immune response. For example, i.p. sensitization with wheat proteins induced a specific IgE response with similar IgE-binding epitopes to humans [22], whereas i.p. sensitization with OVA led to more OVA-specific IgE-binding epitopes compared to oral sensitization [23].

Sensitization to food allergens such as peanut or cow’s milk (CM) may occur in the gut with oral sensitization. Over the last few years, several oral food allergy models were established in rodents [24, 25] and are useful for investigating the mechanisms underlying sensitization and clinical reactions to food proteins. For example, Li et al. [26] demonstrated that oral exposure of C3H/HeJ mice to peanut extract (PE) in combination with cholera toxin (CT) induced PE-specific IgE in serum and systemic anaphylactic symptoms upon oral challenge. Alternatively, the skin may also be a route for sensitization to food allergens [27]. For example, in a human study it was found that cutaneous exposure, rather than maternal or infant allergen consumption, led to peanut sensitization [28]. Recently, Spergel et al. [29] started to decipher cutaneous sensitization mechanisms with food allergens in mice. These authors found that epicutaneous (e.p.) sensitization with OVA, in the absence of aluminum hydroxide, resulted in higher antibody levels compared to i.p. administration of OVA with aluminum hydroxide [29], suggesting that e.p. sensitization is a robust sensitization route. Furthermore, Strid et al. [30] reported that an aqueous solution of either peanut allergen or OVA, when applied to abraded skin of mice, induced the production of antigen-specific IgE. Notably, the most effective route of food allergen sensitization varies significantly between mouse strains [15, 31].

Therefore, the route of allergen sensitization is an important and necessary consideration for the use of any relevant animal model of food allergy. Oral sensitization may be required to mimic the effect of digestion and the gut epithelium on sensitization to food proteins. However, it is not yet known which route of sensitization is best to predict the allergenic potency of food proteins in the human population.

## Dose–sensitization relationships

Risk assessment for food allergens does not fundamentally differ from assessing the risk of chemical substances or microbiological agents as they often include similar methodologies [32–34]. In the hazard characterization of food allergens, a qualitative, and, wherever possible, quantitative description of the sensitizing property of a food allergen is made, together with its relationship to dose, where possible. These dose–sensitization data are helpful to classify food allergens by creating threshold values below which the risk of inducing a new food allergy is considered to be negligible or acceptable.

In humans, knowledge on dose–sensitization relationships to food allergens is extremely limited. Probably both low- and high- dose tolerance induction may be relevant mechanisms for explaining the fact that just a small percentage of the human population develops food allergy [35]. Since many variables (e.g. exposure route and frequency, food-related factors, host-related factors, matrix effects) are likely to be of importance, the doses required for sensitization might prove highly variable.

No animal model has been adopted for use in the generation of dose–sensitization data. Ideally, an appropriate animal model should be (a) validated by dose–response curves with different sensitizations and (b) be sensitive for distinguishing a threshold beyond which significant sensitization would be predicted and (c) potentially be sensitive for producing graded responses comparable to what is known regarding their prevalence and severity of responses in humans, e.g. peanut > egg > spinach [12]. Previously, animal studies demonstrated dose–response relationships within a restricted dose range for only a limited number of proteins [12, 13, 36]. Using these animal studies one can only conclude that there is a hazard connected to a given protein, because the mechanistic knowledge to interpret the dose–sensitization profile in terms of risk assessment is lacking. This was nicely illustrated by Kroghsbo et al. [36] where dose–sensitization data of two related proteins (gluten and enzymatically hydrolysed gluten) were compared to determine which protein is the strongest sensitizer. Enzymatically hydrolysed gluten gave the highest immune response, which was dose-related. Gluten showed no dose-related responses. However, in contrast to the hydrolysed gluten, gluten showed a response at the low dose. Thus, one can conclude that both forms of this protein possess sensitizing capacity and the doses relevant for human exposure should be taken into consideration when qualifying the potential risks for humans.

In conclusion, dose–sensitization studies in animals can be used to enhance our mechanistic knowledge on the sensitization process and characterize the allergenic hazard of novel food proteins. However, the current lack of dose–sensitization data in food allergy makes it difficult to perform a risk assessment. In addition, dose-dependent effects on immunological responses are not always linear, which further complicates interpretation.

## Protein preparation

Ideally, an animal model should assess the sensitizing capacity of the individual novel proteins, as well as the novel protein in the context of the whole food. However, the choice of how the proteins are prepared prior to sensitization assessment may have significant implications on the predictive value of the model. Should purified proteins, protein extracts or the complex whole food be used as test materials? Could the use of whole foods predict the sensitizing potential of individual proteins? Will purified proteins fold into the correct structure in the absence of the food matrix? Are there matrix proteins that modify (potentiate or inhibit) sensitizing capacity? These are only a few of the considerations that should be addressed before choosing a predictive animal model. Studying the sensitizing capacity of an allergen, as a constituent of different protein preparations is a major task that requires well-conducted and controlled animal studies [37].

For novel foods where there is no prior knowledge of the potential allergenicity of proteins contained herein, whole food allergenicity assessment might be the only option to identify potential de novo sensitizing proteins. The use of whole foods has the advantage of presenting the proteins to the immune system in the context of lipids, sugars and other proteins, and matrix factors known to influence the sensitizing capacity of a given protein [37, 38].

When using protein extracts, proteins may be lost or the relative amounts may be changed during the extraction process [37], because extraction is dependent on protein solubility and may be influenced by the processing of the foods [39]. This could result in the testing of an incomplete panel of proteins.

Additionally, the purity and quality of purified proteins must be of a high standard, because the predictive value of the animal model may be greatly influenced by contaminants. Both protein and endotoxin contamination can confuse allergenicity assessments. This issue was highlighted following the use of commercially “purified” OVA, where contamination with ovomucoid (OVM) resulted in an overestimation of the intrinsic sensitizing potential of OVA [40],. This indicates that the presence of small amounts of a potent sensitizer may obscure the sensitizing capacity of the intended study protein. Immune-modulating effects occur with endotoxin contamination, which may potentially lead to an overestimation of the protein-specific sensitizing capacity [41]. Thus, purified proteins should ideally be free of all possible modifying contaminants.

## Protein processing

Foods are subjected to a wide variety of different processing methods before being consumed. Processing may affect the inherent allergenicity of the proteins contained within the food, by either decreasing or increasing their allergenic properties [39, 42]. However, there are no general rules on how and to what degree different forms of processing impact the allergenic properties of the food [39, 42].

Processing methods that affect the allergenic properties of food include heating, hydrolysis, pH and pressure treatment, which may modify the chemical and structural features of the proteins. The impact of heating on the sensitizing capacity of peanut was described by Ladics et al. [12] who compared the sensitizing capacity of raw and roasted peanut extract and observed no clear differences after oral or i.p. dosing of BN rats. Additionally, Bowman and Selgrade [13] showed similar results after oral administration to C3H/HeJ mice. In contrast, Kroghsbo et al. [37] demonstrated that oral dosing of BN rats with roasted peanut, but not whole blanched peanut, resulted in Ara h 1- and 2-specific IgE responses. These studies show that heating intensity can influence the sensitizing capacity of peanut proteins.

Hydrolysis usually reduces allergenicity, however, a study by Kroghsbo et al. [36] showed that acid hydrolysis of gluten proteins resulted in a significantly higher specific IgE response than unmodified gluten, in contrast to enzymatically hydrolyzed gluten, after i.p. immunization of BN rats. In vitro digestion abolished the sensitizing capacity of the CM protein BLG, but the same procedure did not affect sensitization to the peanut protein Ara h 1, even though Ara h 1 was digested to smaller peptides than BLG [9]. These studies collectively showed that hydrolysis may affect individual proteins differently and that the type of hydrolyses may affect the outcome.

## Food matrix

Foods are composed of proteins, fat, carbohydrates, micronutrients and various contaminants, all of which may have various effects on intrinsic allergenicity of proteins by changing protein digestibility, bioaccessibility and/or bioavailability, or due to adjuvant or immune-modulatory effects. These factors should be considered in the in vivo allergenicity assessment of new proteins/protein sources.

Various food constituents can alter the digestibility of proteins, thus affecting the form and the way they will reach the site where immune responses are induced. This can simply result from a buffering effect of the whole food or from the presence of protease inhibitors. Additionally, emulsion of protein with lipids will modify their structure and the accessibility of enzymes to cleavage sites, with various effects on digestibility [43]. Similar effects were observed for added constituents such as stabilizers, thickeners or emulsifiers [44]. Competitive effects of other proteins for enzymatic digestion and active epithelial transport can also impact allergen digestibility and bioavailability [45]. Sequestration of protein in low accessible substructures, such as within protein body organelles as observed in seeds, can delay their release and limit their digestion [46]. High fat food increases gastric residence in humans, thus leading to an increased threshold for the occurrence of objective symptoms [47].

Proteins can co-localize with other food constituents such as pro-Th2 or modulating factors, whereby the corresponding microenvironment will determine the polarization of the specific immune response. Some studies have reported a lack of intrinsic immunogenicity/allergenicity of certain major allergens from milk, peanut or Brazil nuts [48–50]. The immune response was prompted by the adjuvant effect of other food constituents, as demonstrated by (defatted) extract from peanut [48] or with lipids from Brazil nuts that will activate iNKT cells to produce IL-4 [50]. Other proteins (lectins) or contaminants such as aflatoxin present in the food matrix influence sensitization [51], whereas ω-3 PUFA-derived metabolites decrease mast cell activation [52].

Lastly, the quantity of any new protein(s) in food items should be considered. For example, the newly expressed protein Cry1Ab (MON810 maize) was demonstrated to be highly immunogenic when administered as a purified protein, but no Cry1Ab-specific immune response was evident after experimental sensitization with maize flour, probably due to the low levels of Cry1Ab within the flour [50].

## Adjuvants

T cell sensitization to allergenic proteins requires fully activated professional antigen presenting cells (APC) that not only present relevant peptides in the context of MHCII, but also express a range of costimulatory signals [53]. Importantly, the lack of appropriate costimulatory signals results in anergy or tolerance. Substances that can induce costimulation are considered adjuvants, being defined as components that are able to potentiate and/or modulate adaptive immune responses.

It is not well understood to what extent adjuvants are needed to promote an allergic response, but adjuvant signals appear crucial at least in a range of animal studies. Adjuvants influence both the activation and subsequent migration of dendritic cells (DCs) to a draining lymph node, which reside in the vicinity of the first exposure site to potential allergens. It is increasingly realized that signals coming from epithelial cells can instruct DC to become activated APC. These epithelium-derived signals together are referred to as a danger associated molecular pattern (DAMP) and include innate cytokines and chemokines or alarmins [54–56]. Together with a range of immune cells such as innate lymphoid cells, intraepithelial lymphocytes (IELs) [57–59], eosinophils and mast cells, DAMPs determine the outcome of the immune response. The importance of the epithelial barrier in controlling Th2 immune responses has been reviewed more extensively elsewhere [56, 60].

In animal models of food allergy, sensitization by the i.p. route with the use of aluminum hydroxide as an adjuvant is common [61]. The mechanisms behind the adjuvant effect of aluminum hydroxide are still not fully understood [62], but stimulation of DC antigen presentation [63] and a IL-4-driven Th2 response have been described [64]. Additionally, changes in specific antibody responses to aluminum hydroxide-adsorbed antigens have been observed [62, 65, 66] and is probably due to the modulation of antibody responses related to structural changes of the antigens [67].

One of the best known mucosal adjuvants used to sensitize animals to food proteins is CT [26]. The adjuvant effect of CT depends on CD11b DCs and cAMP [68]. Importantly, because *Vibrio cholera* infection is relatively rare in humans, CT should be regarded as an experimental model adjuvant and is not relevant for promoting food allergy in man.

Additional modulating substances may influence sensitization to food proteins. *Staphylococcus aureus* enterotoxin B (SEB), a bacterial superantigen relevant to humans, promotes sensitization to OVA [69]. The NSAID diclofenac causes epithelial damage in the intestinal tract and stimulates the allergic response to peanut extract, but only in combination with CT [57, 58]. Medium chain triglyceride (MCT), but not long-chain triglycerides induce sensitization to peanut in mice, without CT [70]. The role of endotoxin as a possible adjuvant remains unclear because data are not consistent across different experimental models and doses [16, 71]. Uric acid is a DAMP produced by epithelial cells and administration of monosodium urate can replace CT as an adjuvant [72].

Occasionally, allergy in test animals can be induced without adjuvant. Birmingham et al. [73] and Gonipeta et al. [74] sensitized mice to hazelnut and milk whey protein, by transdermal application of the allergen, for 6 consecutive weeks. Although they did not add an adjuvant, they clipped the hair from skin and used mild occlusion for 1 day, which may cause mild inflammatory responses and release DAMPs [75]. Others [54, 76], have used tape stripping methods to promote epicutaneous sensitization to food allergens. Noti et al. [54] showed that this route of exposure requires the TSLP-basophil axis, indicating activation of innate immune responses. Guinea pigs have also been used as a model to investigate the allergenicity of specifically CM without adding adjuvant by exposing the guinea pigs to the CM via their drinking water for several weeks [77]. However it is difficult to translate this model to the human setting due to differences in immune physiology and limited knowledge and tools to study their immune system. Lastly, the BN rat model for food allergy is performed without added adjuvant, but in this model the allergen is gavaged for 35 to 42 days [78], again possibly inducing epithelial stress (in the oesophagus) with associated adjuvant effects.

In conclusion, primarily based on mouse data, adjuvants or at least adjuvant-like activation of innate immunity seems to be important for the induction of sensitization to food proteins. However, addition of an adjuvant will not always be necessary in an animal model when testing sensitizing capacity of novel proteins, especially when the novel protein/food has inherent sensitizing capacity.

## Environmental factors

Not everyone becomes allergic to foods. This suggests that other factors like lifestyle and environmental factors, interacting with a genetic predisposition, play a role. To accurately predict the allergenic potential of novel food proteins using animal models, it is essential to consider the various environmental factors that could influence sensitization in humans.

Firstly, unintended dietary pre-exposure to the food protein under investigation or to a cross-reactive protein could lead to the induction of allergen-specific oral tolerance, which would prevent further sensitization in the animal model and lead to false negative results. Dietary control in parental generations before mating or during suckling [79, 80] and monitoring other dietary factors such as the quantity of bioactive lipid components or non-digestible fibers in animal diet, which influence the immune response, can help minimize potential bias in sensitization profiles [81–83]. However, other currently unknown dietary factors, may also influence immune responses within the gut and further research is needed to identify these factors.

Protein modifications (e.g. due to environmental pollution or during food processing) have a substantial impact on the elicitation of protein-specific immune responses. In addition, interference with the physiological digestion capacity of the GI tract contributes to food allergy. Pharmacological gastric acid suppression is associated with food allergy development via the oral route in experimental mouse models [84, 85]. While animal age seems to play a minor role in many models [86], the use of newborn/weaned animals can be relevant when using a different experimental approach to induce sensitization or if the protein being investigated is ultimately intended for consumption by human infants [87].

The composition of the gut microbiome may influence the outcome of food allergy models and may contribute to inter-laboratory variation. There is increasing evidence that gut microbiota plays a critical role in allergic sensitization and tolerance induction in humans and rodents [88, 89]. The fetal immune system favors a Th2 response that is related to an increased risk of developing allergic disease. Bacterial colonization after birth provides a microbial stimulus affecting the maturation and modulation of the intestinal and systemic immune system [90]. Commensal bacteria can stimulate tight junction-related proteins thereby reducing epithelial permeability, while also promoting immunoregulatory responses within mucosal tissue which protects against allergic sensitization [91–94]. Germ-free mice display a characteristic increased immune response to allergens with a remarkable Th2 bias. Thus, these animals could represent a highly sensitive model to study allergenicity of new proteins [95, 96], but are difficult to maintain. The gut microbiome of different animal facilities will be influenced by the breeding environment (e.g. specific pathogen free (SPF) versus specific and opportunistic pathogen free (SOPF), diet and water). In particular, microbiome alterations associated with ω-fatty acids and obesity should be controlled. Lastly, there are indications that vitamin A and D deficiencies, which modify intestinal homeostasis, might moderate intestinal immunity via interaction with the microbiome [79, 97].

Breeding environment and experience of the experimenter should be taken into consideration, since stress responses may influence the immune response to the administered protein/food [98]. In conclusion, breeding conditions (parental generations, housing, stress), diet and other environmental factors must be carefully adjusted between different laboratories and standardized whenever possible to develop a reproducible animal model to study protein sensitization. Unfortunately, many published manuscripts still do not describe these parameters in detail and therefore, currently, it is not possible to recommend a specific dietary regimen, other than the protein of interest should not be included in the diet before testing the animals.

## In vivo readouts

Common food allergy signs and symptoms in patients include itching, swelling of lips, tongue, face and throat, abdominal pain, diarrhea, nausea, or vomiting, while anaphylactic reactions involve constriction of airways, cardiovascular shock with a severe drop in blood pressure, rapid pulse and/or loss of consciousness [99]. Upon exposure to food allergens, a number of allergy signs that mimic clinical symptoms in patients can be observed in animal model systems [3, 100]. Such in vivo parameters are useful to study allergenicity of food proteins, the impact of genetics or microbial colonization [101]. To model food allergy, animals are typically sensitized with an allergenic food or protein (with or without adjuvant) by feeding or other routes, followed by challenges to the GI tract, circulation (intravenous (i.v.), i.p.), or skin (subcutaneous), which then manifests in an organ-specific distinct readout.

Repeated oral food allergen challenges of previously sensitized animals results in measureable clinical signs including diarrhea, piloerection, changes in activity, mobility and behavior or most often a combination of all signs that can be enumerated in a clinical allergy score [54].

Systemic food allergen challenges often result in severe allergic reactions mimicking anaphylaxis in patients. Such reactions are evaluated using anaphylaxis scoring protocols that assess severity including scratching, diarrhea, piloerection, labored respiration, cyanosis around mouth and tail, reduced activity, tremors, convulsion or death [26]. Measuring hypothermia (rectal temperature or subcutaneously (s.c.) implanted programmable temperature transponder) or vascular leakage (i.p allergen challenge immediately followed by i.v. Evan’s blue injection and animals are monitored for blue color accumulation in the extremities) are additional in vivo readouts [102, 103].

To measure airway hyperreactivity (AHR) in the context of food allergen sensitization, allergen challenge may be intranasal, intra-tracheal or via nebulization [104]. Upon allergen challenge, animals are assessed for airway resistance and compliance in response to methacholine and not by allergen exposure using invasive or enhanced pause (PenH) non-invasive techniques [105]. Notably, this read out may be more dependent upon changes in the airways (e.g., inflammation and increased airway smooth muscle) than to the mast cell-IgE-histamine axis.

Passive cutaneous anaphylaxis (PCA) is an immediate dermal response to an allergen-IgE interaction that is typically characterized by increased vascular leakage within the skin that can be assessed by i.v. injection of Evans blue. In vivo PCA readouts include ear swelling (thickening of skin) and skin color [103]. Alternatively animals are injected intradermally (i.d.) in the ear pinnae with the allergen and ear swelling is measured within 1 h. This acute allergic skin response can be used to asses an immediate type hypersensitivity (ITH) [106, 107]. Delayed type hypersensitivity (DTH) represents an additional skin test to assess late-phase cutaneous food allergic reactions, in which animals are injected s.c. with allergen into the hind footpads or in the ear pinnae and edema measured [108].

While in vivo readouts allow for a rapid assessment of allergic responses, a caveat of these readouts is that measures of allergy are often subjective and thus, require blinding of experimental groups. In vivo readouts provide more information than just sensitization potential as allergy effector mechanisms become activated, although not all of these responses are IgE-dependent. Lastly, one should ensure that ethical concerns are considered, particularly when inducing severe allergic reactions. The advantages and disadvantages of the different in vivo readouts are summarized in Table 1.

**Table 1:** In vivo readouts

Test	Advantages	Disadvantages
Gastrointestinal [54]	Non-invasive, does not harm animals, qualitative and quantitative allergy scoring, blinded scoring possible	No standardized scoring system, lab to lab variations, subjective, diarrhea as only GI specific sign
Systemic		
Anaphylaxis score [76]	Non-invasive, qualitative and quantitative allergy scoring, blinded scoring possible	Subjective, ethical consideration
Hypothermia [109, 110]	Rectal temperature (semi-invasive), quantitative readout, blinded scoring possible	Accuracy of rectal measurements, transplanted responders (invasive), ethical consideration
Vascular leakage [24, 110]	Qualitative readout, blinded scoring possible	Invasive
Airways		
AHR [105]	Qualitative and quantitative, objective readout, blinded measurements are possible, anesthesia not required for non-invasive AHR	Invasive and anesthesia required (only for invasive AHR), usually endpoint measurement, expensive equipment required
Skin [111]		
PCA	Quantitative measurement of skin thickness, qualitative assessement of vascular leakage	Invasive, blinded scoring not possible
ITH	Quantitative measurement of skin swelling	Invasive, blinded scoring not possible
DTH	Quantitative measurement of skin/tissue swelling	Invasive, blinded scoring not possible

## Ex vivo readouts

A wide range of ex vivo readouts can be utilized to assess or support the sensitizing capacity of novel proteins. The most common readout consists of measuring specific IgE antibody levels from exposed animals by ELISA [112]. However, allergen-specific IgG may obscure the analysis as allergen-specific IgG generally is present at 100 to 1000-fold higher concentrations than allergen-specific IgE. IgE detection can be improved by depleting IgG or by employing a capture ELISA [48, 113, 114]. In addition, new technologies such as rapid evanescent biosensor technology would be useful to avoid the influence of IgG when measuring allergen-specific IgE [115]. Although total IgE may be correlated with specific IgE in controlled experimental settings [116], identification of allergen-specific IgE is required for allergenic assessment of novel foods. It is also important to assess the biological activity of antigen-specific IgE. For example, functionality of serum IgE may be assessed in vitro as the ability to induce specific degranulation of basophils or mast cells [117].

Allergic sensitization starts with activation of innate cells, including epithelial cells, DCs and ILC2 s, T cells and Th2 cytokines [118, 119]. Cytokine production and immune cell proliferation are typically measured after ex vivo stimulation of cells from the lymph node or spleen with the allergen or with T cell mitogens [113, 120]. While intracellular cytokine production on the single cell level can be determined by flow cytometry, cytokine secretion from cell suspensions is measured as supernatant concentrations by ELISA or multiplex assays. The Th1/Th2/Th17/Treg cytokine balance, rather than the absolute cytokine levels, is thought to be important [121]. Current models suggest that cytokine and proliferation responses during the induction phase of sensitization in the draining lymph node may be useful in predicting sensitizing capacity [122–124]. Measuring TSLP, IL-25 and IL-33, along with ILC2 s, in the intestine during food allergy sensitization may provide additional predictive markers of sensitizing potential [26, 55].

Determination of cell phenotypes, subsets and co-stimulatory molecules on innate and adaptive immune cells in the lymph node, spleen or intestines are readouts possibly useful to support a sensitizing potential. Such measurements can be performed by high throughput flow cytometric or mass spectrometry-based assays. Determination of gene expression (e.g. mRNA) and cytokine gene epigenetics, co-stimulatory molecules or inflammatory markers are also ex vivo endpoints currently applied.

Many animal models for food allergy investigate the anaphylactic response to a food allergy challenge [24, 76, 107, 110]. Ex vivo endpoints for anaphylaxis include serum mast cell proteases (mMCP-1), [125] and histamine release assays.

## Future perspectives and conclusions

Considerable progress has been made in using animal models to better understand the basic mechanisms and environmental influences contributing to food allergen sensitization. Researchers intending to utilize animal models of food allergy should be aware of the experimental parameters outlined in this review, which may have an impact on their results. In addition, published reports should include sufficient details concerning all of these parameters, to allow for reproduction in other laboratories. The Pros and Cons of the experimental parameters discussed in this review are summarized in Table 2. The ideal animal model for assessing the potential sensitizing capacity of new proteins has yet to be developed, but the ideal model must predict known strong and weak food allergens. The development of a reference protein toolbox is essential and would revolutionize the use of animal models in the future risk assessment of potential allergens. Ideally, the sensitization route would be oral or via the skin and not only IgE measurements, but also functional or symptomatic responses should be recorded. In addition, more research is required to determine why only some proteins are allergenic in contrast to the majority of proteins. The identification of certain protein characteristics such as structural similarities or intrinsic activities will greatly assist the development of animal models for the screening of allergenic potential. However, even known food allergens do not induce food allergy in all exposed individuals and therefore, host and environmental factors also need to be explored further, which can be achieved through the use of animal models.

**Table 2:** Pros and Cons of different food allergy model design parameters

Design parameter	Sub-parameter	Pros	Cons
Reference proteins	Strong, weak and non-allergenic proteins	Confirm reproducibility and predictability	Requires additional groups of animals
Animal species	Mice	Small size, short breeding cycle, availability of many reagents	Usually need adjuvants, low amount of sera can be obtained
	Rats	Small size, short breeding cycle, larger amount of sera can be obtained, no need for use of adjuvant	Restricted availability of reagents, larger amount of protein/food required
	Dogs	Large organ size and increased amount of sera can be obtained, spontaneously develop allergies	Restricted availability of reagents, Very large amount of protein/food required, large animals, prolonged duration of animal studies, expensive and ethical consideration
Route of sensitization	Oral	Relevant route of sensitization	Often needs the use of adjuvant, require large amount of protein
	i.p.	No adjuvant, robust sensitization route, no need for large amount of proteins/food	Non-physiological relevant route
	Cutaneous	Relevant route of sensitization	Usually requires immunological danger signals
Dose–response relationship		Helps in creating threshold levels, helps in hazard identification	Several groups of animals required for each protein
Protein preparation	Whole foods	Ability to study the sensitizing capacity of proteins in their natural matrix, Ability to study the allergenicity of true novel foods, Easy to prepare	Difficult to identify the sensitising proteins
	Purified proteins	Ability to study the inherent sensitizing capacity of the individual protein	Difficult to prepare, need large amounts of high quality purified protein, protein structure may change
	Food Extracts	Easy to prepare	Difficult to identify the sensitizing protein, some proteins may be lost or the relative amounts may change, protein structure may change
Protein processing	Raw protein/food	Ability to study the inherent allergenicity	May not reflect the end use of the protein
	Processed protein/food	Ability to study the sensitizing capacity of the consumed version which cannot be predicted otherwise	May impact the allergenic properties
Adjuvant		Provides a danger signal	Artificially modifies the immune response

## Abbreviations

APC: antigen presenting cell
BLG: beta-lactoglubulin
BN: Brown Norway
CM: cow’s milk
CT: cholera toxin
DAMP: danger associated molecular pattern
DC: dendritic cells
DTH: delayed type hypersensitivity
e.p.: epicutaneous
i.d.: intradermally
IEL: intraepithelial lymphocytes
IL: interleukin
ILC: innate lymphoid cell
i.n.: intranasal
iNKT: invariant natural killer T cell
i.p.: intraperitoneal
ITH: immediate type hypersensitivity
i.v.: intravenous
LTP: lipid transfer protein
MCT: medium chain triglyceride
OVA: ovalbumin
OVM: ovomucoid
PCA: passive cutaneous anaphylaxis
PE: peanut extract
SEB: staphylococcus aureus enterotoxin B
SOPF: specific and opportunistic pathogen free
SPF: specific pathogen free
Th: T helper cell
TSLP: thymic stromal lymphopoietin
